# Medical Fees

**Published:** 1911-02-04

**Authors:** 


### The Hospital

A Journal of

The Medical Sciences and Hospital Administration.

New Series. No. 209, Vol. VIII. [No. 1281, Vol. XLIX.] SATURDAY FEBRUARY 4 1911.

### Medical Fees.

Discussions on the subject of the fees of medical practitioners recur periodically in the public Press, and little that is new has been revealed by the recent correspondence in the *Times*. An historical research into the question would bring many interesting facts to light, not the least noteworthy being that since the earliest times there have always been people who complained of the size of the doctor's bill, however moderate, and deferred its payment until long after the accounts of the butcher, the baker, and the candlestick-maker had been settled. Concerning this question, William Wadd, surgeon extraordinary to King George IV., collected much entertaining material, which was published in his "Mems and Maxims" in 1827. He cites a conversation which passed between the physician Bouvart and a French Marquis, whom he had attended during a long and severe indisposition. As he entered the sick chamber on a certain occasion, he was thus addressed by his patient: "Good day to you, Mr. Bouvart, I feel quite in spirits, and think my fever has left me." "I am sure of it," replied the doctor, "the very first expression you used convinces me of it." "Pray explain yourself." "Nothing more easy; in the first days of your illness, when your life was in danger, I was your *dearest friend*; as you began to get better I was your *good Bouvart*; and now I am Mr. Bouvart; depend upon it, you are quite recovered." Unfortunately, as the writer observes, the practitioner's remuneration comes when the impressions of fear, hope, and gratitude are almost effaced, and payment is made with indifference, hesitation, reluctance, and reproach.

There are some who quarrel with the principle of regulating fees according to the patient's ability to pay, notwithstanding that the custom has prevailed for at least four thousand years. The practice existed, in fact, among the physicians of ancient Babylon, and was sanctioned by the legal scale of physicians' fees in the Code of Hammurabi, who was King of Babylon about 2250 B.C. The fee for an important operation "with a bronze lancet" was from two to ten shekels of silver, according to the status of the patient—if the operation were successful. But it would not be altogether wise to insist too much on the justice of the law relating to medical practice in ancient Babylon, for it provided that the surgeon whose operation on a member of the upper class was unsuccessful should have his fingers cut off. Here, again, the sliding scale took effect, for if the patient was the slave of a freeman, and died under the operation, the surgeon had to restore a slave of equal value. Possibly the Chinese system, or what is reputed to be the Chinese system, of "no cure no pay" is open to less objection. In Portugal it used to be no uncommon thing to pay the physician by presenting him with his own portrait, but the faculty have met with rougher treatment, and it is recorded that Austrigilda, wife of Gontram, King of Burgundy, had, in compliance with her dying request, her two physicians slain and buried with her. As Wadd quaintly observes, these were probably the only two medical gentlemen who were ever privileged to lie in the tombs of kings. A doubtful honour of another kind was bestowed upon the chief physician to Pope Adrian; the night after the Pope's decease the door of the physician was ornamented with garlands, with the inscription, "To the deliverer of his country."

The most handsome fee mentioned by Wadd was that paid to Baron Dimsdale for inoculation of the Empress of Russia and her son; he was made a Baron of the Empire, with a present of £12,000, and a pension of £500 a year. Dr. Willis, for his successful attendance on King George III., was rewarded by £1,500 a year for twenty years, and £650 a year to his son for life. An example of a fee at the other end of the scale is to be found in the account of the expenses of the Earl of Cumberland, in the seventeenth year of Henry VIII.; thus, "Item to a physician at Westminster, for seying mr Lord's water, 4d." In the Court of the Kings of Wales the physician or surgeon was partly paid in kind; for curing a flesh wound he was allowed such of the garments of the wounded person as were stained with blood, but for curing a dangerous wound he was allowed a hundred and eighty pence and his maintenance, besides the blood-stained garments. At the end of the seventeenth century the usual fees to physicians and surgeons were, according to Wadd, who quotes a book called "Levamen Infirmi" as his authority: "To a graduate in physick, his due is about ten shillings, though he commonly expects or demands twenty. Those that are only licensed physicians, their due is no more than six shillings and eightpence, though they commonly demand ten shillings. A surgeon's fee is twelve pence a mile, be his journey far or near; ten groats to set a bone broke, or out of joint; and for letting blood, one shilling; for cutting off, or amputation, of any limb, is five pounds; but there is no settled price for the cure." It is highly probable that this scale of charges was not strictly adhered to.

As to the question of stating items in rendering accounts, there is an abundance of evidence that this used to be customary. It may be interesting to refer to one or two old doctors' bills. The first is one made out by Dr. Isaac Dobell, who practised in the parish of Cranbrook, Kent, and his client was a Mr. Relf, who lived a few miles from the practitioner's house. The bill is dated 1780, and the items are as follows: "October 1: To visit to his daughter Mary, 1s.; to cathartic mix., 1s. October 2: to visit to do., 1s.; to ℥iv. saline and anodyne mix., 1s. 6d.; to paper of cathartic powd., 3d. October 3: to visit to do., 1s. October 6: to visit, 1s.; to paper of cathartic powd., 3d. Total, 7s." Another of Dr. Dobell's bills, dated 1782, shows that sometimes his charge for a visit was only 6d., but by 1794 his fee had risen to 1s. 6d. When the patient lived at a considerable distance the fees were naturally higher, and a bill made out to a patient who lived fifteen miles from Dr. Dobell's house contains the item "journey, 7s. 6d.," while another bill, dated 1798, contains the item "To a delivery, 10s. 6d." These fees are not necessarily typical of the charges made by practitioners at that time, as could be shown by reference to other bills. The truth is, to quote Wadd once more, "a liberal mind will always be influenced by the condition of the patient; nor is there any profession in which liberality more abounds, or in which so much gratuitous service is rendered to suffering humanity, as in the honourable profession of medicine."

